# Transposable element detection from whole genome sequence data

**DOI:** 10.1186/s13100-015-0055-3

**Published:** 2015-12-29

**Authors:** Adam D. Ewing

**Affiliations:** Mater Research Institute - University of Queensland, 37 Kent St Level 4, Woolloongabba, QLD 4102 Australia

**Keywords:** Methods, Sequencing, Bioinformatics

## Abstract

The number of software tools available for detecting transposable element insertions from whole genome sequence data has been increasing steadily throughout the last ~5 years. Some of these methods have unique features suiting them for particular use cases, but in general they follow one or more of a common set of approaches. Here, detection and filtering approaches are reviewed in the light of transposable element biology and the current state of whole genome sequencing. We demonstrate that the current state-of-the-art methods still do not produce highly concordant results and provide resources to assist future development in transposable element detection methods.

**Electronic supplementary material:**

The online version of this article (doi:10.1186/s13100-015-0055-3) contains supplementary material, which is available to authorized users.

## Background

It has been 27 years since Haig Kazazian, Jr. published the seminal observation of active LINE-1 retrotransposition in humans [1], and 14 years since the initial publication of the assembled human genome reference sequence gave us a genome-wide view of human transposable element content, albeit largely from one individual [2]. Because LINEs, Alus, and SVAs are actively increasing in copy number at estimated rates of around 2-5 new insertions for every 100 live births for Alu [3–5], and around 0.5-1 in 100 for L1 [4–7], it stands to reason that the vast majority of transposable element insertions are not present in the reference genome assembly and are detectable as segregating structural variants in human populations.

**Fig. 1:**
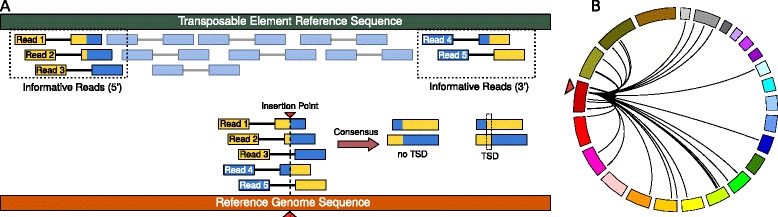
Read mapping patterns typically associated with insertion detection. Panel **a** shows the read mapping patterns versus a reference TE sequence (grey rectangle, top) and the mapping of the same reads to a reference genome sequence (orange rectangle, bottom). Reads are represented as typical paired-end reads where the ends of each amplicon are represented as rectangles and the un-sequenced portion of the amplicons are represented as bars connecting the rectangles. Reads informative for identifying TE insertion locations are indicated by dashed boxes, other read mappings to the TE reference are shown in light blue boxes. Within the informative reads, reads or portions of reads mapping to the TE reference are coloured blue, and mappings to the reference genome sequence are coloured yellow. The exact location of this example insertion is indicated by the red triangle and the dashed line. Assembly of the reads supporting the two junction sequences is indicated to the right of the ‘consensus’ arrow, one example with a TSD and one without. If a TSD is present, the insertion breakends relative to the reference genome are staggered, and the overlap of reference-aligned sequence corresponds to the TSD. If a TSD is not present (and no bases are deleted upon insertion), the junctions obtained from the 5' end and the 3' end of the TE reference will match exactly. Panel **b** shows a typical pattern of discordant read mappings across a genome - the colored segments in circle represent chromosomes, each black link indicates a discordant read mapping supporting an insertion at the position indicated by the red triangle. The endpoints not corresponding to the insertion site map to TE elements at various locations in the reference genome

Identification of transposable element insertions (TEs) from the results of currently available high-throughput sequencing platforms is a challenge. A number of targeted methods are available to sequence junctions between TEs and their insertion sites, and have been reviewed elsewhere [8–10]. Similarly, there are several methods used for transposable element identification and annotation from genome assemblies, also reviewed elsewhere [11–15]. This review focuses on methods for discovering and/or genotyping transposable elements from whole genome sequence (WGS) data. The majority of the WGS data available today comes from Illumina platforms and consists of millions to billions of 100-150 bp reads in pairs, where each read in a pair represents the end of a longer fragment (Fig. 1a). Detection of small mutations, single-base or multiple-base substitutions, insertions, and deletions less than one read length, is achievable through accurate alignment to the reference genome followed by examination of aligned columns of bases for deviations from the reference sequence. Detection of structural variants is more difficult, principally because using current whole genome sequencing methods, the presence of rearrangements versus the reference genome must be inferred from short sequences that generally do not span the entire interval affected by a rearrangement. Typically, structural variant detection from short paired-end read data is solved through a combination of three approaches: 1. inference from discordant read-pair mappings, 2. clustering of ‘split’ reads sharing common alignment junctions, and 3. sequence assembly and re-alignment of assembled contigs [16].

Transposable elements represent a majority of structural insertions longer than a few hundred base pairs [17], and require a further level of scrutiny on top of what is normally required for SV detection, which is informed by their insertion mechanism. This review is principally concerned with the detection of non-Long Terminal Repeat (LTR) retrotransposons in mammalian genomes, but many of the concepts should generalise to other transposable element types in other species. Regarding the mechanism of insertion, non-LTR retrotransposition in mammals is driven by the activity of Long INterspersed Elements (LINEs) which replicate through an mRNA-mediated series of events known as target-primed reverse transcription (TPRT) [18]. There are a number of important features of TPRT which one must be cognisant of when devising methods for detecting retrotransposon insertions. First, a message must be transcribed, and it seems that 3' polyadenylation is a necessary feature for recognition by poly-A binding proteins associated with the L1 Ribonuclear Particle (RNP) [19–22]. This does not necessarily mean that the message must be Pol II transcribed: for example, Alu elements are Pol III transcripts [23]. Insertions are processed transcripts: the cultured cell retrotransposition assay relies on this fact, as there is an intron in reverse-orientation to the reporter gene in these assays, which is spliced out when the construct is transcribed [24]. Additionally, the detection of processed pseudogenes uses the presence of splice junctions between coding exons as a defining feature [25, 26]. Polyadenylation at the 3' end of inserted L1 and SVA sequences is generally observed, and shorter A tails also exist on the 3' end of Alu insertions.

Target-site duplication (TSD) is a feature of TPRT that is necessary to consider when detecting novel insertions. The ORF2 endonuclease cleavage is staggered, meaning there is some distance, typically 7-20 base-pairs [27], between the cut sites in the top strand and bottom strand. Some software tools have been developed specifically to detect TSDs [28, 29]. Once the insertion site is fully resolved at the end of TPRT through mechanisms that likely include host DNA repair but are incompletely understood, the sequence between the cut sites appears on either site of the new insertion. Although insertions without TSDs do occur due to co-occurring deletions at the target site (about 10 % of insertions) [30, 31], or via the endonuclease-independent pathway [32], the vast majority of new insertions occurring through TPRT have TSDs, and these can generally be readily identified through sequence analysis methods when identifying novel insertions.

Insertion of transduced sequences is another feature of transposable element insertions that may be detected computationally and is important to consider when applying or designing methods for insertion detection. When sequences immediately adjacent to the transposable elements are transcribed up- or down-stream as part of the TE message, both the TE RNA and non-TE RNA will be reverse transcribed and integrated into the insertion site as a DNA sequence [33–35]. As LINE insertions are often 5' truncated [36, 37], sometimes transduced sequences are all that is left of a message with a severe 5' truncation. As a result, in some instances an insertion may contain no recognizable transposable element sequence, but the mechanism can be surmised from the presence of the poly-A tail and TSDs [38].

Roughly 1 in 5 LINE insertions will have an inversion of the 5' end of the element due to a variant of the TPRT mechanism known as ‘twin-priming’, where two ORF2 molecules reverse-transcribe the L1 RNA from different directions, resulting in an insertion with a 5' end inversion. [39]. This is an important consideration when designing methods to identify insertions of these sequences, as the relative orientation of the 5' end is not predictable and filtering putative insertion sites without taking this into account may lead to a 20\% higher false negative rate for LINE detection from the 5' end.

Finally, maybe the most important feature of transposable element insertions that impacts methods used for their detection is simply their repetitive nature in the context of the reference genome: due to repeated copy-and-paste operations through TPRT, there are thousands of elements from each active class of transposable element present in the human genome. This is the key factor that makes accurate detection of transposable element insertions difficult: read pairs mapping to the insertion site will have paired ends that map to various locations throughout the reference genome where instances of the inserted element type are present (Fig. 1b). The presence of many copies of an element in the genome also confounds detection of new copies of that element by introducing false positives where what appears to be a novel insertion may actually just be a mapping artefact of an existing transposable element present in the reference genome.

## Review

Given whole genome sequence (WGS) data, there are three basic approaches to looking for non-reference insertions that are often used together, integrating support from each approach: discordant read-pair clustering, split-read mapping, and sequence assembly. It bears mentioning that all of these are not applicable to every WGS method; read-pairs are not necessarily present depending on the library preparation method or sequencing technology. Currently, the most widespread approach to WGS is via Illumina HiSeq technology using paired-end reads. In the future, as methods for long-read sequencing mature, new computational methods for insertion detection may be required, or previous methods for detecting insertions from capillary sequence or comparative whole-genome assemblies [4] may be repurposed.

## Discordant read-pair mapping

A discordant read pair is one that is inconsistent with the library preparation parameters. During library preparation, genomic DNA is sheared physically or chemically, and fragments of a specific size are selected for library preparation and sequencing. Given an expected fragment size distribution, anything significantly outside of that range may be considered discordant. What is significantly outside of the expected range of fragment sizes can be determined after sequencing and alignment based on the distribution of distances between paired reads. Additionally, given the library prep method and sequencing platform, the expected orientation of the ends of the read-pairs is known. For instance, Illumina read pairs are ‘forward-reverse’ meaning that relative to the reference genome, the first read in a pair will be in the ‘forward’ orientation and the second will be ‘reverse’. Reads inconsistent with this pattern may be considered discordant. Finally, reads pairs where one end maps to a different chromosome or contig than the other are considered discordant.

When using discordant read pairs to inform structural variant discovery, typically multiple pairs indicating the same non-reference junction must be present. For events between two regions of unique mappable sequence such as chromosome fusions, deletions, duplications, etc. the locations of both ends of the collection read pairs supporting an event should be consistent. As transposable elements exist in many copies dispersed throughout the genome, typically one end will be ‘anchored’ in unique sequence while the other may map to multiple distal locations located within various repeat elements throughout the genome (Fig. 1b). In general, there are two approaches to analysing discordant reads where one end maps to repeat sequence. One is to map all reads to a reference library of repeats, collect the reads where only one end in the pair aligns completely to the reference repeat sequences, and re-mapping the non-repeat end of these one-end-repeat pairs to the reference genome (Fig. 1a). A second approach is to use the repeat annotations available for the reference genome to note where one end of a pair maps to a repeat and the other does not (Fig. 1b). In either case, once ‘one-end-repeat’ reads have been identified, the non-repeat ends of the read pairs are clustered by genomic coordinate, and possibly filtered by various criteria concerning mapping quality, consistency in read orientations, underlying genomic features, and so forth. For example, TranspoSeq filters calls where greater than 30 % of clustered reads have a mapping quality of 0 [40], while Jitterbug excludes reads with a mapping quality score of less than 15 [41]. Most tools filter out insertion calls within a window around transposable element annotations in the reference genome. It is important to note that discordant read mapping alone does not yield exact junctions between the insertion and the reference sequence, therefore sites localised by discordant read mapping are typically refined through local sequence assembly and split-read mapping.

## Split-read mapping

Split reads are where one segment maps to some location in the reference genome, and the remaining segment maps to one or more locations distal from the first, or is unmapped (i.e. does not match anything in the reference). This term may also refer to a longer assembled contig which can be split into multiple mapped locations distal from one another. The ability to detect split reads is highly dependent on the choice of aligner. Some short read aligners (e.g. BWA MEM [42]) have the ability to partially align (‘soft’ or ‘hard’ clip) reads and give alternate mapping locations for the clipped portion as secondary or supplementary alignments. Aligners intended for lower throughput and longer reads (BLAT [43], LAST [44], BLAST [45]) are natural choices for detecting split reads, especially from longer assembled sequences. Since split reads are the means for identifying the exact insertion location at base-pair resolution, analysis of split reads is critical for identifying features indicative of TPRT activity including transductions, target site duplications, endonuclease cleavage site, and the addition of untemplated bases. Additionally, it is possible to take advantage of overlaps between reads supporting an insertion and use sequence assembly in an attempt to generate longer contigs of sequence that better resolve the junctions between the insertion and the reference genome, essentially creating very long split reads which have the potential to span both the 5' and 3' junctions of an inserted sequence. This is particularly useful for elucidating transduced sequences and studying untemplated base incorporation at the junctions in detail. In general, it is highly advisable that TE detection methods incorporate split-read analysis as this is the primary means to detect 5' and 3' junctions with nucleotide resolution, and thus the primary means to detecting many hallmarks of TE insertion necessary both for filtering false positives and for biological inferences.

## Filtering putative insertions

Given the challenge associated with detecting structural variants from short-read data, compounded with the difficulty of detecting insertions of sequences into a background that already contains thousands of similar interspersed copies, any scheme purporting to detect transposable element insertions with reasonable sensitivity must implement filters to control for false positives.

Most methods use the number of reads supporting an insertion as a first cutoff - either as a parameter or as a function of local sequence depth. For WGS data, split reads and discordant read support may be considered independently when filtering insertions. The target allele fraction (i.e. fraction of cells in which an insertion is expected to be present) is an important consideration: somatic insertions arising later in the history of a tissue or a tumour may be supported by fewer reads than germline insertions expected to be present in 1-2 copies per mononucleated cell. In addition to the quantity of reads, the quality of reads should be considered both in terms of their alignment and base quality. Base quality (e.g. phred score) over clipped bases is particularly important when considering soft clipped read mappings: if the clipped bases have poor quality, it is likely they do not represent transposable element sequence and can be ignored. Mappings of high-quality sequence with a high number (e.g. > 5 %) of mismatches versus either the genome around the insertion site or versus the consensus transposable element are often associated with false positives, but this cutoff should be implemented according to the expected divergence of the TE insertions with respect to the reference TE sequence: if the available TE reference is not a good representation of the expected insertions (e.g. the reference is constructed from a different species) this filter should be relaxed.

A second major consideration when filtering transposable element insertions is the nature of the genome at the insertion site. As with any attempt at annotation or mutation detection versus a reference genome, the concept of mappability (or alignability) is important [46, 47]. A sequence is considered ‘mappable’ (or ‘alignable’) if it aligns to one and only one location. For a given segment of the reference genome, mappability can be calculated by considering the number of uniquely mapping k-mers (i.e. sequences of length *k*) corresponding to commonly encountered read lengths (e.g. 35 bp, 50 bp, 100 bp), possibly allowing for some number of mismatches. Filtering insertions that overlap annotated transposable elements is often done and may serve as a proxy for mappability as TE sequences often have relatively fewer unique k-mers relative to the non-repeat genome.

As mentioned, it is usually advisable to filter TE insertions that map onto the coordinates of TEs of the same subfamily represented in the reference genome. This is due to low mappability over recent transposable element insertions due to their similarity to the active consensus element, which can be addressed using a mappability filter as described, and it also guards against artefacts due to similarity between the insertion site and the inserted element. Finally, in instances where the goal is detection of somatic or novel germline insertions, a good database of known non-reference insertion sites is essential. Existing published resources to this end include dbRIP [48] and euL1db [49]. As the former has not been updated in some years and the latter only considers L1 insertions, a simple listing of reported non-reference insertion coordinates derived from the supplementary tables associated with most current studies reporting non-reference human retrotransposon insertions is included as Additional file 1: Table S1 (see Addtitional file 1 for table legend).

## Considerations for analyses in non-humans

Many of the methods listed in Table 1 have been successfully applied to species other than human, and to transposable element varieties other than the non-LTR elements focused on in this review so far. For example Retroseq [50] has been applied to mouse genomes to detect LTR elements such as IAP and MusD in addition to the mouse varieties of LINE (L1Md) and SINE (B1/B2) elements [51]. T-lex [52] and T-lex2 [53] have been applied to *Drosophila* genomes, detecting a wide variety of different TE families. While non-LTR TEs in human have a consensus insertion site preference that is widespread in the human genome, other TE families have more specific integration site preferences. For example, the Ty1 LTR retroelement strongly prefers integration near Pol III transcribed tRNA genes and seems to associate with nucleosomes [54], while Tf1 elements (also LTRs) prefer nucleosome-free regions near Pol II promoters [55]. Hermes elements (a type of DNA transposon) also prefer nucleosome-free regions and have a characteristic TSD sequence motif (nTnnnnAn) [56]. Non-LTR retroelements can also have strong insertions site preferences as well, a prominent example being the R1 and R2 elements from *Bombyx mori*, which target 28S ribosomal genes [57] and have been used to dissect the biochemical steps involved in non-LTR integration [18]. These various propensities to insert proximal to genomic features and have defined sequence characteristics at the insertion site could be used to filter insertion detections from WGS data for these TE families in non-human species, in combination with the general approaches already covered for non-LTR elements that have weaker insertion site preferences. Additionally, some of the characteristics of non-LTR retrotransposition presented so far may not apply to other TE classes and families and could lead to false negatives if putative insertions are inappropriately filtered against certain characteristics. For example, some DNA transposons (e.g. Spy) do not create target site duplications, so software that requires TSD will miss these [58]. Other TEs have fixed TSD lengths, e.g. the Ac/Ds transposons in maize, famously initially described by McClintock in the 1950s [59], create an 8 bp TSD [60, 61], so a detector that allows Ac/Ds predictions with other TSD sizes might be more prone to false positives.

**Table 1:** Software for detecting transposable element insertions from WGS data

Name of method	Detection target	Ref.	Notes or use case	Implementation	Availability
TranspoSeq	Transposable elements	[40]	Analysis of Tumour/Normal WGS pairs, extension to analyse WES data as well	Java, R	https://www.broadinstitute.org/cancer/cga/transposeq
Tea	Transposable elements	[65]	Analysis of Tumour/Normal WGS Pairs	R	http://compbio.med.harvard.edu/Tea/
TraFiC	Transposable elements	[66]	Analysis of Tumour/Normal WGS Pairs, detection of transduced sequences	Perl	https://github.com/cancerit/TraFiC
RetroSeq	Transposable elements	[50, 51]	Used for analysis of mouse strain genomes, also demonstrated on human, has genotyping and discovery modes	Perl	https://github.com/tk2/RetroSeq
Tangram	Transposable elements	[75]	Demonstrated on 1000 Genomes Project samples, includes genotyping capability	C, C++	https://github.com/jiantao/Tangram
VariationHunter	Structural Variants	[76, 77]	Among the first methods to detect polymorphic Alu insertions from WGS	C++	http://compbio.cs.sfu.ca/software-variation-hunter
GRIPper	Retrotransposed mRNAs	[78]	Used to detect non-reference gene retrocopy insertions. Demonstrated in humans, mice, and chimpanzees.	Python	https://github.com/adamewing/GRIPper
T-lex/T-lex2	Transposable elements	[52, 53]	Detects both insertions versus the reference and absences of reference elements in other genomes. Demonstrated on Drosophila TEs.	Perl	http://petrov.stanford.edu/cgi-bin/Tlex.html
HYDRA-SV	Structural rearrangements	[79]	General-purpose SV detection, also detects TE insertions	C++, Python	https://github.com/arq5x/Hydra
RelocaTE	Transposable elements	[80]	Demonstrated on mPing insertions in Oryza sativa (rice)	Perl	https://github.com/srobb1/RelocaTE
ITIS	Transposable elements	[81]	Used to detect Tnt1 insertions in Medicago truncatula	Perl	http://bioinformatics.psc.ac.cn/software/ITIS/
ngs_te_mapper	Transposable elements	[82]	Requires TSDs, demonstrated in Drosophila	R	https://github.com/bergmanlab/ngs_te_mapper
TE-Locate	Transposable elements	[83]	Used to examine TE insertions in Arabidopsis populations	Java, Perl	http://sourceforge.net/projects/te-locate/
TIGRA	Structural rearrangements	[84]	Assembly-based SV detection method, demonstrated to identify TE breakpoints	C++	https://bitbucket.org/xianfan/tigra
Mobster	Transposable elements	[85]	Demonstrated on WGS and WES data, Illumina and ABI SOLiD data.	Java	http://sourceforge.net/projects/mobster/
TEMP	Transposable elements	[86]	Geared towards population-level TE detection from pooled data	Perl	https://github.com/JialiUMassWengLab/TEMP
TE-Tracker	Transposable elements	[87]	Attempts to determine source elements in reference. Demonstrated on Arabidopsis.	Perl	http://www.genoscope.cns.fr/externe/tetracker/
Jitterbug	Transposable elements	[41]	Demonstrated on Human and Arabidopsis.	Python	http://sourceforge.net/projects/jitterbug/
DD_DETECTION	Transposable elements	[88]	Does not require input of canonical TE sequences (Database-free)	C++	https://bitbucket.org/mkroon/dd_detection
MELT	Transposable elements	[89]	Used for comprehensive analysis of 2504 participants in the 1000 Genomes Project	Java	http://melt.igs.umaryland.edu/

## Comparing methods

When it comes to detecting mutations, especially somatic mutations, different methods and/or different parametrisations yield markedly different results [62–64], and transposable element detection is no exception [5]. Publications presenting new tools often include comparisons where a number of competing methods are run by the authors of the new tool. While valuable, these experiments may not reflect optimal parametrisations of the competing tools for the dataset used as a basis of comparison, whereas by virtue of having developed a novel method, the authors will have better parametrisations of their own tools, leading to the usual outcome of the new tool outperforming previously published methods.

**Fig. 2:**
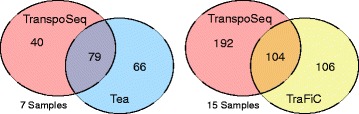
Intersections between somatic insertion detection methods. Overlap and non-overlap between insertion sites from reported in publications using the indicated software tools on the same data. The number of samples included in the comparison shared between the each pair of studies is indicated

To illustrate the extent of the differences in TE insertion calls from different methods run on the same data, we present comparisons between somatic TE detections from three recent studies. In each case, two different methods were used to call mutations on the same data, yielding substantial overlap and an equally if not more substantial amount of non-overlap. Importantly, these calls were generated by the developers of their respective TE detection methods. Coordinates and sample identities were obtained from the supplemental information of the respective studies, and one [65] needed to be converted from hg18 to hg19 coordinates via liftOver. Insertion coordinates were padded by +/- 100 bp and compared via BEDTools v2.23. Lee et al. [65] (Tea) and Helman et al. [40] (TranspoSeq) share 7 samples, Tubio et al. [66] (TraFiC) and Helman et al. (TranspoSeq) share 15 samples. No samples are shared between Lee et al. and Helman et al. The overall Jaccard distance between TranspoSeq and Tea results across shared samples was 0.573 (Additional file 2 and Additional file 3: Table S2a), and between TranspoSeq and TraFiC the distance was 0.741 (Additional file 2 and Additional file 3: Table S2b), indicating that TranspoSeq and Tea seem to yield more similar results than between TranspoSeq and TraFiC. Summing counts for intersected insertion calls and method-specific calls yields the overlaps shown in Fig. 2. While this comparison is somewhat cursory and high-level, it is clear there is a substantial amount of difference in the results of these methods: in both comparisons, more insertions are identified by a single program than by both programs. Given that all three studies report a high validation rate (greater than 94 %) where samples were available for validation, this may reflect a difficulty in tuning methods for high sensitivity while maintaining high specificity. This also suggests that perhaps an ensemble approach combining calls across all three (or more) methods may be preferable where high sensitivity is required.

In addition to the tools already highlighted, a rapidly increasing number of tools exist with the common goal of detecting transposable element insertions from WGS data. As indicated in Table 1, these include purpose-built methods aimed specifically at transposable elements in addition to more general methods that identify a wide variety of structural alterations versus a reference genome, transposable element insertions included. Table 1 is not intended to represent an exhaustive listing of currently existing methods - the OMICtools website (http://omictools.com/) currently supports an up-to-date database of TE detection tools, and the Bergman lab website also hosts a list of transposable element detection tools which includes tools aimed at a wide variety of applications, a subset of which are relevant for TE detection from WGS data [11].

## Conclusions

Transposable element insertions are a subset of structural variants that can be identified from WGS data. Although generalised SV discovery methods sometimes support TE detection, specialised software is often used by those interested in studying the specific peculiarities of the insertion mechanism and mitigating the false positives associated with their high copy number. TE discovery methods developed in the last 5 years are predominantly aimed at short-read paired-end WGS data, most often generated on Illumina platforms, and use a combination of paired-end, split-read, and sequence assembly approaches to identify insertions. Technological and methodological developments will change how the ascertainment of transposable element insertion sites is carried out. Long-read sequencing has the potential to both improve resolution of TE insertions, especially those located in repetitive regions [67], and to improve the information available regarding the sequence of the insertion itself. Currently this technology has been successful for *de novo* assembly of microbial genomes [68], but for human genomes, high sequence coverage [69] and a combination multiple sequencing approaches [70] and sophisticated error correction models [71] may be required to get a good consensus sequence given the currently high error rates associated with long-read sequencing technologies. Over time, it is expected that throughput will increase and error rate will decrease, making this a viable option. Even if relatively higher error rates for long-read single-molecule sequencing approaches persist, the key may be to obtain good whole-genome assemblies of individual genomes accomplished through higher throughput. Methodologically, new software tools will be published when new sequencing technologies or new alignment methods and formats attain widespread acceptance. Additional new software tools utilising current sequencing technology will also continue to be developed and published - that said, it is important that new methods offer some demonstrable, substantial improvement over the many existing methods, and there does appear to be room for improvement given the low concordance currently observed between different tools on the same data. For those seeking to develop additional methods, an improved focus on software engineering and usability would also be welcome. The subfield of transposable element insertion detection from WGS data currently lacks standards against which authors of new tools can benchmark their methods. Some recent tools have been tested on high-coverage trios e.g. NA12878/NA12891/NA12892 which is probably a step in the right direction as these are high-quality and readily available. Establishing or extending standardised datasets such as those already developed for variant calling [72, 73] would be a further step in the right direction. Going beyond this, a “living benchmark” similar to what exists for protein structure prediction through CASP [74] or more topically what currently exists through the ICGC-TCGA DREAM Somatic Mutation Calling Challenge [64] would provide a publicly available “proving ground” for existing and novel TE insertion detection methods.

## Additional files

Additional file 1:
**Supplemental Table Legends.** Description and references for supplemental tables 1 and 2. (PDF 48 kb) Additional file 2: Table S1. List of known or predicted germline insertions derived from several publications. (BED 1239 kb) Additional file 3: Table S2. Sample-level examination of intersections between TE detection methods. (XLSX 6 kb)

## Abbreviations

L1: LINE-1/Long Interspersed Element-1
LTR: Long Terminal Repeat
RNP: Ribonuclear Particle
SV: Structural Variant
SVA: SINE VNTR ALU
TE: Transposable Element
TPRT: Target-primed Reverse Transcription
TSD: Target Site Duplication
VNTR: Variable Number of Tandem Repeats
WGS: Whole Genome Sequencing
