# Supplementary material for: Transposable element detection from whole genome sequence data
Source: Mob DNA. 2015 Dec 29;6:24. doi: 10.1186/s13100-015-0055-3 (PMC4696183; doi:10.1186/s13100-015-0055-3)
Supplement: Additional file 1: — Supplemental Table Legends. Description and references for supplemental tables 1 and 2. (PDF 48 kb) [file 13100_2015_55_MOESM1_ESM.pdf]

Supplementary Table Legends for "Transposable element detection from whole genome sequence data"

Adam D. Ewing (adam.ewing@mater.uq.edu.au)

September 2, 2015

1 Supplementary Tables

1.1 Supplementary Table 1

Text file containing the following columns: Chromosome, Start, End, TE Family (L1, ALU, or SVA), and a comma-delimited list of studies, abbreviated as follows: AE2010[1], AE2011[2], AK2014[3], CB2010[4], CS2011[5], DBRIP[6], DW2010[7], DW2013[8], EH2014[9], EL2012[10], FH2011 [11], JT2014[12], RI2010[13], RS2013[14], SS2012[15]. Where coordinates were originally relative to hg18, they were converted to hg19 using the liftOver tool. The final list was generated using BEDTools merge.

1.2 Supplementary Table 2

Overlaps between insertion sites identified by *(a)* TranspoSeq and Tea or *(b)* TranspoSeq and TraFiC, related to Figure 2. Each row shows the overlap for an individual sample, and the summation shown in Figure 2 is the bottom row for each panel. Jaccard distance between two insertion sets A and B is defined as $1 - \frac{|A \cap B|}{|A \cup B|}$.

2 Supplementary References

References

[1] Ewing, A.D., Kazazian, H.H.: High-throughput sequencing reveals extensive variation in human-specific L1 content in individual human genomes. Genome Res. **20**(9), 1262–1270 (2010). doi:10.1101/gr.106419.110

[2] Ewing, A.D., Kazazian, H.H.: Whole-genome resequencing allows detection of many rare LINE-1 insertion alleles in humans. Genome Res. **21**(6), 985–990 (2011). doi:10.1101/gr.114777.110

[3] Kuhn, A., Ong, Y.M., Cheng, C.-Y., Wong, T.Y., Quake, S.R., Burkholder, W.F.: Linkage disequilibrium and signatures of positive selection around LINE-1 retrotransposons in the human genome. Proc. Natl. Acad. Sci. U.S.A. **111**(22), 8131–8136 (2014). doi:10.1073/pnas.1401532111

[4] Beck, C.R., Collier, P., Macfarlane, C., Malig, M., Kidd, J.M., Eichler, E.E., Badge, R.M., Moran, J.V.: LINE-1 retrotransposition activity in human genomes. Cell **141**(7), 1159–1170 (2010). doi:10.1016/j.cell.2010.05.021

[5] Stewart, C., Kural, D., Strmberg, M.P., Walker, J.A., Konkel, M.K., Sttz, A.M., Urban, A.E., Grubert, F., Lam, H.Y.K., Lee, W.-P., Busby, M., Indap, A.R., Garrison, E., Huff, C., Xing, J., Snyder, M.P., Jorde, L.B., Batzer, M.A., Korbel, J.O., Marth, G.T., 1000 Genomes Project: A comprehensive map of mobile element insertion polymorphisms in humans. PLoS Genet. **7**(8), 1002236 (2011). doi:10.1371/journal.pgen.1002236

[6] Wang, J., Song, L., Grover, D., Azrak, S., Batzer, M.A., Liang, P.: dbRIP: a highly integrated database of retrotransposon insertion polymorphisms in humans. Hum. Mutat. **27**(4), 323–329 (2006). doi:10.1002/humu.20307

[7] Witherspoon, D.J., Xing, J., Zhang, Y., Watkins, W.S., Batzer, M.A., Jorde, L.B.: Mobile element scanning (ME-Scan) by targeted high-throughput sequencing. BMC Genomics **11**, 410 (2010). doi:10.1186/1471-2164-11-410

[8] Witherspoon, D.J., Zhang, Y., Xing, J., Watkins, W.S., Ha, H., Batzer, M.A., Jorde, L.B.: Mobile element scanning (ME-Scan) identifies thousands of novel Alu insertions in diverse human populations. Genome Res. **23**(7), 1170–1181 (2013). doi:10.1101/gr.148973.112

[9] Helman, E., Lawrence, M.S., Stewart, C., Sougnez, C., Getz, G., Meyerson, M.: Somatic retrotransposition in human cancer revealed by whole-genome and exome sequencing. Genome Res. **24**(7), 1053–1063 (2014). doi:10.1101/gr.163659.113

[10] Lee, E., Iskow, R., Yang, L., Gokcumen, O., Haseley, P., Luquette, L.J., Lohr, J.G., Harris, C.C., Ding, L., Wilson, R.K., Wheeler, D.A., Gibbs, R.A., Kucherlapati, R., Lee, C., Kharchenko, P.V., Park, P.J., Cancer Genome Atlas Research Network: Landscape of somatic retrotransposition in human cancers. Science **337**(6097), 967–971 (2012). doi:10.1126/science.1222077

[11] Hormozdiari, F., Alkan, C., Ventura, M., Hajirasouliha, I., Malig, M., Hach, F., Yorukoglu, D., Dao, P., Bakhshi, M., Sahinalp, S.C., Eichler, E.E.: Alu repeat discovery and characterization within human genomes. Genome Research **21**(6), 840–849 (2011). doi:10.1101/gr.115956.110

[12] Tubio, J.M.C., Li, Y., Ju, Y.S., Martincorena, I., Cooke, S.L., Tojo, M., Gundem, G., Pipinikas, C.P., Zamora, J., Raine, K., Menzies, A., Roman-Garcia, P., Fullam, A., Gerstung, M., Shlien, A., Tarpey, P.S., Papaemmanuil, E., Knappskog, S., Van Loo, P., Ramakrishna, M., Davies, H.R., Marshall, J., Wedge, D.C., Teague, J.W., Butler, A.P., Nik-Zainal, S., Alexandrov, L., Behjati, S., Yates, L.R., Bolli, N., Mudie, L., Hardy, C., Martin, S., McLaren, S., O'Meara, S., Anderson, E., Maddison, M., Gamble, S., ICGC Breast Cancer Group, ICGC Bone Cancer Group, ICGC Prostate Cancer Group, Foster, C., Warren, A.Y., Whitaker, H., Brewer, D., Eeles, R., Cooper, C., Neal, D., Lynch, A.G., Visakorpi, T., Isaacs, W.B., van't Veer, L., Caldas, C., Desmedt, C., Sotiriou, C., Aparicio, S., Foekens, J.A., Eyfjrd, J.E., Lakhani, S.R., Thomas, G., Myklebost, O., Span, P.N., Brresen-Dale, A.-L., Richardson, A.L., Van de Vijver, M., Vincent-Salomon, A., Van den Eynden, G.G., Flanagan, A.M., Futreal, P.A., Janes, S.M., Bova, G.S., Stratton, M.R., McDermott, U., Campbell, P.J.: Mobile DNA in cancer. Extensive transduction of nonrepetitive DNA mediated by L1 retrotransposition in cancer genomes. Science **345**(6196), 1251343 (2014). doi:10.1126/science.1251343

[13] Iskow, R.C., McCabe, M.T., Mills, R.E., Torene, S., Pittard, W.S., Neuwald, A.F., Van Meir, E.G., Vertino, P.M., Devine, S.E.: Natural mutagenesis of human genomes by endogenous retrotransposons. Cell **141**(7), 1253–1261 (2010). doi:10.1016/j.cell.2010.05.020

[14] Shukla, R., Upton, K.R., Muoz-Lopez, M., Gerhardt, D.J., Fisher, M.E., Nguyen, T., Brennan, P.M., Baillie, J.K., Collino, A., Ghisletti, S., Sinha, S., Iannelli, F., Radaelli, E., Dos Santos, A., Rapoud, D., Guettier, C., Samuel, D., Natoli, G., Carninci, P., Ciccarelli, F.D., Garcia-Perez, J.L., Faivre, J., Faulkner, G.J.: Endogenous retrotransposition activates oncogenic pathways in hepatocellular carcinoma. Cell **153**(1), 101–111 (2013). doi:10.1016/j.cell.2013.02.032

[15] Solyom, S., Ewing, A.D., Rahrmann, E.P., Doucet, T., Nelson, H.H., Burns, M.B., Harris, R.S., Sigmon, D.F., Casella, A., Erlanger, B., Wheelan, S., Upton, K.R., Shukla, R., Faulkner, G.J., Largaespada, D.A., Kazazian, H.H.: Extensive somatic L1 retrotransposition in colorectal tumors. Genome Res. **22**(12), 2328–2338 (2012). doi:10.1101/gr.145235.112
